# Evolving eukaryotes: an interview with Joel Dacks

**DOI:** 10.1186/s12915-018-0586-4

**Published:** 2018-11-01

**Authors:** Joel B. Dacks

**Affiliations:** 1 Faculty of Medicine and Dentistry, University of Alberta, 5-31 Medical Science Building, Edmonton, Alberta Canada; 2 Department of Life Sciences, The Natural History Museum, Cromwell Road, London, SW7 5BD UK

**Keywords:** Evolution, Genomics, Eukaryotes, Membrane-trafficking, Golgi, Parasitism

## Abstract

Joel Dacks is an Associate Professor and Canada Research Chair in Evolutionary Cell Biology at the University of Alberta, a Scientific Associate at the Natural History Museum (London), and the current President of the International Society for Evolutionary Protistology. His research group studies the evolution and diversity of the eukaryotic membrane-trafficking system, from origins to potential disease therapeutics. In this interview, Joel shares some perspectives on gaining a balanced view of comparative cell biology and the importance of a constructive peer review process.

## What are your current research interests?

My lab studies the evolution and diversity of membrane-trafficking organelles. Using genomics and molecular evolutionary techniques, with a focus on microbial eukaryotes (protists), we are examining the conservation in how material is moved around the cell and the ways in which different lineages across eukaryotes vary from the canonical configuration. This information tells us about the diversity of modern membrane trafficking mechanisms and function. It can also be used to understand the evolutionary processes and details of how the endomembrane organelles, and their associated molecular machinery, arose [1]. Currently, we have several projects examining the genomes of parasitic lineages (and their harmless free-living relatives) to understand the evolution of the specialized membrane-trafficking machinery that underpins parasitic mechanisms [2]. We also have a project using transcriptomics and bioinformatics to investigate the gene regulatory networks and cell biology of contractile vacuoles, endolysosomal organelles that regulate osmotic pressure in freshwater protists. Finally, we are delving into the evolution of key membrane-trafficking families such as SNAREs (e.g., [3]), vesicle coats (e.g., [4]), and GTPases (e.g., [5]).

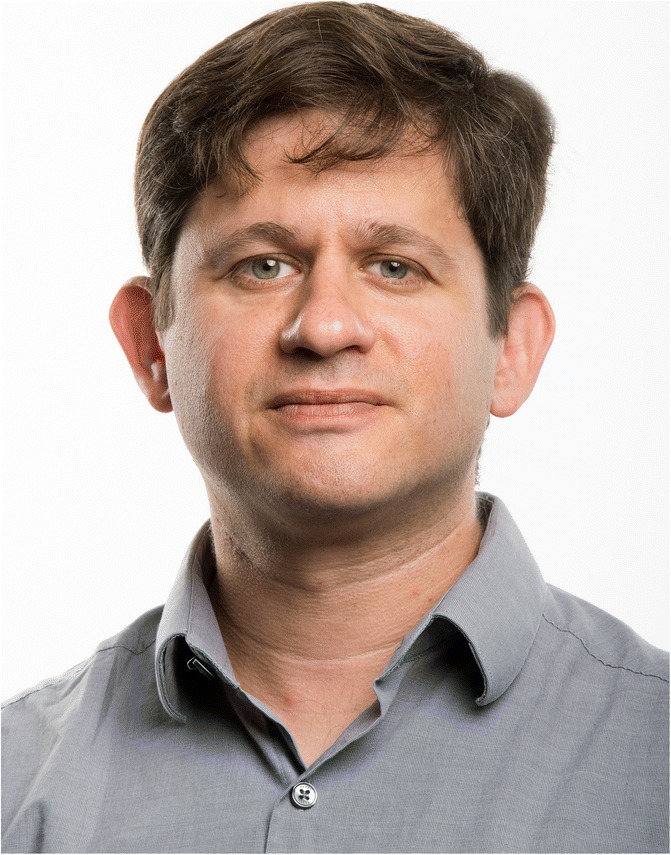



## What are your predictions for the field over the next 5 years?

There has been a major community effort to produce tractable molecular cell biological model systems in the areas of the eukaryotic tree other than plants, animals, and fungi (e.g., the MMI program [6]). I expect that these efforts are going to increasingly produce a wealth of comparative data, enabling a more balanced and informed view of all of eukaryotic cell biology. Also, the field of eukaryogenesis was revolutionized in the past few years by the discovery of the Asgard archaea as the closest prokaryotic relatives of eukaryotes [7]. Efforts to isolate and culture these organisms will lead to even greater advances in understanding both a previously unknown type of organism and the contributors to eukaryogenesis. I also hope that new lineages will be described which are even closer to eukaryotes than the Asgard archaea already described.

## What motivates you to provide peer review for journals?

I think that the peer-review process is more important now than ever. With the vast amount of material available online that can be used to inform and mis-inform, a rigorous peer-review process is absolutely crucial since it is what sets scientific data apart from opinion backed by anecdotal facts. As well, we as a community rely on the good-will and sense of responsibility of our colleagues to keep the peer-review system working. My colleagues do this, and I feel a responsibility to contribute.

## Have you had any memorably good or bad experiences of peer review, as an author or as a reviewer?

My two best peer-review experiences, in retrospect though perhaps not at the time, were both rejections. The first was a rejection of a paper I submitted whilst in graduate school, where the editor told me that the result was interesting but that I needed to collect more data to test my interpretation of the findings that we had. But the next data that we collected were seemingly contradictory to the first! So we kept collecting and looking at different data points to bring into the study. Because we had to keep pushing and piece together data that were more complex than initially anticipated, we ended up coming up with a very different paper, one that laid the foundation for our proposed mechanism of how non-endosymbiotically derived eukaryotic organelles evolve. The second experience was quite recent. We had submitted our work to several high impact journals and received form-letter rejections. Finally, we got a rejection but with detailed and thoughtful criticism. It turned out that the reviewers were interpreting our paper in a way that I had not anticipated, prompting me to rewrite the entire manuscript to clarify our points. The next submission to a different high impact journal was completely straightforward and positive.

### Website:

http://www.chairs-chaires.gc.ca/chairholders-titulaires/profile-eng.aspx?profileId=2871.
